# A Social Model of Loneliness: The Roles of Disability, Social Resources, and Cognitive Impairment

**DOI:** 10.1093/geront/gnw125

**Published:** 2016-11-09

**Authors:** Vanessa Burholt, Gill Windle, Deborah J Morgan, CFAS Wales team

**Affiliations:** 1 Centre for Innovative Ageing, College of Human and Health Science, Swansea University, UK; 2 Dementia Services Development Centre, Bangor Institute of Health & Medical Research, Bangor University, UK

**Keywords:** Social interaction, Social model of disability, Isolation, Dementia, Analysis—moderated-mediation modeling

## Abstract

**Purpose of the study:**

We consider the points at which cognitive impairment may impact on the pathway to loneliness for older people, through impeding social interaction with family and friends, or by interfering with judgments concerning satisfaction with relationships.

**Design and methods:**

We conceptualize a mediation model anticipating that social resources (LSNS-6) will mediate the pathway between disability (Townsend Disability Scale) and loneliness (De Jong Gierveld 6-item scale) and a moderated-mediation model in which we hypothesize that cognitive impairment (MMSE) will moderate the association between disability and social resources and between social resources and loneliness. To validate the hypothesized pathways, we draw on the CFAS Wales data set (*N* = 3,593) which is a nationally representative study of community-dwelling people aged 65 and older in Wales.

**Results:**

Disability had a significant indirect effect on loneliness through the mediating variable social resources. Cognitive impairment was significantly associated with social resources, but did not moderate the relationship between disability and social resources. Cognitive impairment had a significant impact on loneliness, and moderated the effect of social resources on loneliness.

**Implications:**

Social structures can (dis)empower people with cognitive impairment and lead to exclusion from social resources or impact on the social construction of aging, cognitive impairment, and dementia. The sense of self for an older person with cognitive impairment may be influenced by social norms and stereotypes, or through a temporal social comparison with an “earlier” sense of self. We conclude that loneliness interventions should be theoretically informed to identify key areas for modification.

This article draws on cognitive discrepancy theory to hypothesize a pathway from disability to loneliness in later life. Reflecting the complex relationship between disability and loneliness, the article takes into account the mediating and moderating effects of the social environment and cognitive impairment. A majority of studies exploring the association between social resources, loneliness, and cognitive function examine the potential for negative cognitive health outcomes. As an alternative to this approach, we consider the different points at which cognitive impairment may impact on the pathway to loneliness for older people—that is, through impeding social interaction with family and friends, or by interfering with judgments concerning satisfaction with relationships. We describe how the cognitive discrepancy theory (Perlman & Peplau, 1998) can be used to situate our hypotheses concerning the “social” role of cognitive impairment in loneliness.

In this article, we use the term “social resources” to refer to the quantity and quality of interactions with family, friends, and neighbors. We also use “social interaction” to refer to ways in which people communicate with each other, and “social network” to describe the configuration of active social relationships. “Loneliness” describes a negative and unpleasant emotional state that is the result of dissatisfaction with the quantity and quality of social resources. The “social environment” refers to the sociocultural context in which people live comprising attitudes and values of the people and institutions with which they interact. “Cognitive impairment” is one of the clinical features of dementia and describes a continuum of deteriorating brain function related to aging that can range from no impairment to severe impairment.

## Critical Gerontological and Psychosocial Approaches

Biological and neurological approaches often assess the role of social support and loneliness in terms of the influence on negative health outcomes, such as cognitive impairment or dementia. Thus, from the perspective of a medical model, lower social functioning may be the manifestation of a precursor to dementia (Fratiglioni, Paillard-Borg, & Winblad, 2004;Holwerda et al., 2012). The effect is hypothesized to operate through indirect pathways (e.g., stress hypothesis, vascular hypothesis, and cognitive reserve;Fratiglioni et al., 2004). However, there is some speculation about the interdependent nature of the relationship between cognitive impairment and loneliness (Bosma et al., 2002;Ellwardt, Van Tilburg, & Aartsen, 2015;Hultsch, Hertzog, Small, & Dixon, 1999). Taking a psychosocial or social gerontological perspective the effect of the physical, social and attitudinal environment would also be assumed to influence social participation. One way to examine this is to apply the International Classification of Functioning, Disability and Health (ICF) to the pathway(s) to loneliness (World Health Organization [WHO], 2001).

In the ICF model “capacity” refers to the “ability of the individual in executing tasks in the standard environment” (WHO, 2001, p. 11). An inability to undertake activities of daily living, impacts on individuals’ ability to maintain their usual lifestyles, including customary levels of social interaction (Slivinske, Fitch, & Morawski, 1996). Mild cognitive impairment does not notably interfere with activities of daily living. Therefore, the extent to which capacity is influenced by cognitive impairment and subsequently impacts on performance (social interaction) and loneliness could be due to contextual factors associated with cognitive impairment. The social environment, including ways in which older people with cognitive impairment are treated by others, may in turn influence social exclusion or self-identity and subsequently impact on social behaviors, access to social resources and the expression of loneliness.

### Social Exclusion

Research suggests that the family network is relatively stable from adolescence to old age, but personal and friendship networks decrease throughout adulthood (Wrzus, Hänel, Wagner, & Neyer, 2013). In particular, evidence indicates that networks shrink in the face of functional or cognitive decline (Aartsen, Van Tilburg, Smits, & Knipscheer, 2004). A critical social gerontology perspective focuses on aspects of the social environment such as social status, social exclusion, and discrimination that may account for the lower access to social resources for older people experiencing functional or cognitive decline.

The social functioning of people with early or mild stages of dementia is influenced by the way in which they are treated by family and formal care givers (Sabat & Lee, 2012). However, there is very little research that explores stigma, prejudice, discrimination, and stereotypes associated with cognitive impairment (Harrison, 2014). The proliferation of national policies focusing on the development of dementia friendly, dementia capable, dementia positive, or dementia supportive communities (Lin & Lewis, 2015) acknowledge that inclusion, integration, and equity needs to improve. These policies imply that there are barriers to full social participation for people with cognitive impairment. We draw on this perspective to suggest that public attitudes, stigma, and discrimination may create barriers that influence access to social resources, and may also impact on self-identity.

### Self-identity and Social Comparison

We use the term “self-identity” to refer to role identification developed over the life course (Cohen-Mansfield, Parpura-Gill, & Golander, 2006). Self-identity may be influenced by the stereotypes and negative images of cognitive impairment in the social environment and in turn impact on the process of social comparison, that is the ways in which individuals evaluate their situation by comparing themselves to others (Festinger, 1954).

During the process of cognitive decline, some authors have argued that older people have awareness and insight into their own condition and respond to changes in cognitive function by maintaining or adjusting existing identities or by developing a new sense of self even into most advances stages of dementia (e.g.,Clare, 2003). However, the persistence of self-identity is dependent on social interaction. The extent to which others focus on “dysfunction” rather than “healthy” attributes of a person will influence the construction of self-identity and the extent to which a person engages in fulfilling social activities. Furthermore, social interaction through friendship requires the recollection of names or shared experiences and a failure to remember may lead to withdrawal from social interactions, a loss of self-confidence and modification of self-identity (Parikh, Troyer, Maione, & Murphy, 2015). Upward social comparison may contrast the perceived downward trajectory of social interaction (stereotype) to earlier lifecourse experiences or the social lifestyles of people without cognitive impairment, resulting in loneliness.

In order to undertake social comparison a person is assumed to have self-knowledge with which they judge and evaluate their situation against others (Buunk & Mussweiler, 2001). While we noted above, that a self-identity is maintained in the face of cognitive impairment, this does not preclude cognition impacting on social comparison. Research has estimated that 3% of people with MCI and 42% of people with mild dementia have some form of anosognosia (Orfei et al., 2010). People with anosognosia are unable to acknowledge fully the cognitive and behavioral changes that have occurred (Mograbi, Brown, & Morris, 2009). The inability to update self-referential information in social contexts (Ruby et al., 2007), may lead to a more positive subjective self-image than objective indicators warrant, and we suggest that these may contribute to a mismatch between achieved and desired social interaction (loneliness).

## Cognitive Discrepancy Theory

Perlman and Peplau (1998) developed a discrepancy model of loneliness, which is outlined inFigure 1. Cognitive discrepancy theory suggests that loneliness is a subjective, unpleasant, and distressing phenomenon stemming from a discrepancy between individuals’ desired and achieved levels of social relations. Mismatch is associated with specific circumstances and life events, including migration, widowhood, or the onset disability. For example, poor health impacts on individuals’ ability to maintain customary levels of social interaction (Burholt & Scharf, 2014). Avoiding loneliness entails addressing the mismatch, by adjusting either*expectations* regarding the quality and frequency of social interaction or*achieved* quality and frequency of social interaction to balance both elements. Any discrepancy between desired and achieved social relations may be labeled by an individual as loneliness. However, this does not lead directly (or inevitably) to loneliness. The authors note that loneliness may be modified by the person’s reaction to the situation and suggest that cognitive processes may modulate the experience. To date, research on loneliness has not attended to the sociocultural and social-structural characteristics of the environment (De Jong Gierveld, Van Tilburg, & Dykstra, 2006). We propose to integrate these features into the cognitive discrepancy model as potential influences on actual or desired social relations.

**Figure 1.**
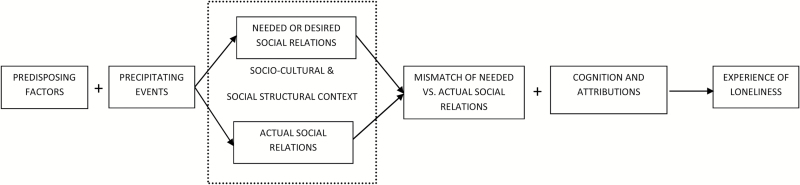
A discrepancy model of loneliness adapted fromPerlman and Peplau (1998).

## Hypotheses

This article draws on cognitive discrepancy theory and builds on the existing evidence regarding associations between individual, psychosocial and social-structural factors to conceptualize a mediation model and a moderated-mediation model that reflects the complex relationship between functional health, cognitive health, social resources, and loneliness (Hayes, 2012a). The conceptual model is followed by empirical validation of the hypothesized pathways using a nationally representative dataset of older people living in Wales.

Factors associated with loneliness (including age, gender, area deprivation, supported living environment, education, and marital status;Ó Luanaigh & Lawlor, 2008;Pitkala & Routasalo, 2003), are controlled for in our models and we treat these as predisposing variables. Disability is the independent variable and primary precipitating event. We hypothesize that greater disability (limitations in activities of daily living) will be associated with greater levels of loneliness (Hypothesis 1). We also hypothesize that disability will have a negative influence on social resources, and decrease the*achieved* level of social interaction (Hypothesis 2). We expect social resources to mediate between disability and loneliness (Hypothesis 3). Further, moving beyond the original components of cognitive discrepancy theory, we hypothesize that cognitive impairment will have an impact on*achieved* levels of social interaction. Specifically, cognitive impairment will moderate the model’s “a” path by amplifying any difficulties associated with accessing social resources because of additional social structural and sociocultural barriers (Hypothesis 4).

Finally, we take into account the desired level of social interaction and an older person’s ability to modify this to match their level of social interaction and avoid feelings of loneliness. We propose that cognitive impairment moderates how intensely people react to levels of social contact and support in two ways. Firstly, we suggest that some people experiencing moderate cognitive impairment will internalize negative stereotypes of cognitive decline and judge that their future will not meet their desires for social interaction, and thus label themselves as lonely. Secondly, we propose that anosognosia in people with greater levels of cognitive impairment may lead to an unrealistic positive expectation of social contact (based on past events), that does not compare to actual levels of social resources, and will also lead to loneliness. Adopting either position, we hypothesize that cognitive impairment will have a moderating influence on the model’s “b” path, and will amplify the influence of disability on loneliness through the mediating variable social resources (Hypothesis 5). To our knowledge, this psychosocial element of the cognitive discrepancy theory has not been tested previously.

## Design and Methods

### Study Sample

We draw on cross-sectional (Wave 1) data (version 2) from the Cognitive Function and Ageing Study (CFAS Wales), a nationally representative study of community-dwelling people aged 65 and older in Wales. Participants were randomly sampled from primary care registration lists in three Local Authorities in Wales, UK: Neath Port Talbot, Gwynedd, and Anglesey. Primary care registration provides the most robust population base for epidemiological studies in the United Kingdom (Matthews et al., 2013). Sampling was stratified according to age group (65–74 years: ≥75 years). Before contacting selected participants, primary care practices records were screened for death, terminal illness or violent behavior.

Interviewers received intensive 3-day training to deliver the standardized interviews (Matthews et al., 2013). Wave 1 interviewing commenced in 2012 and was completed in 2014. Computer-assisted personal interviews were conducted in participants’ homes through the medium of English or Welsh. In total, 3,593 interviews were conducted with participants aged 65 and older. The response rate was 46%. This article is based on a sample of 3,314 participants with no missing data on the variables used in the analysis.

The average age of participants was 74.6 years (SD = 6.97) and they had on average completed 11.7 years (SD = 2.71) of full time education. Participants were predominantly female (*n* = 1,795, 54%), married (*n* = 2,044, 61.7%) and lived in the community rather than in a care setting (*n* = 25, 0.8%). To account for nonresponse, we control for age, gender, care setting, and area deprivation status and use 5,000 bootstrap samples to derive robust estimates of confidence intervals for the coefficients in the models tested below.

### Measures

#### Independent Variable

Limitations in activities of daily living were measured using the Modified Townsend Disability Scale (Bond & Carstairs, 1982;Townsend, 1979). This scale consists of nine activities: cutting own toenails, washing all over or bathing, getting on a bus, going up and down stairs, heavy housework, shopping and carrying heavy bags, preparing and cooking a hot meal, reaching an overhead shelf, and tying a good knot in string. For each activity, participants were assigned a score of 2 if they needed help; 1 if they had some difficulty or used aids; and 0 if they had no difficulty. The scores for each item were summed (range 0–18) and then recoded into a 5-point ordinal variable representing: 0 (no incapacity), 1–2 (slight incapacity), 3–6 (some incapacity), 7–10 (appreciable incapacity), and 11 or more (severe incapacity) (Melzer, McWilliams, Brayne, Johnson, & Bond, 2000)

#### Dependent Variable

Loneliness was measured using the six-item De Jong Gierveld scale. The score is the sum of all items, where higher scores represent greater levels of loneliness. The six-item scale has a reported alpha coefficient of reliability ranging from 0.70 to 0.76 (De Jong Gierveld & Van Tilburg, 2006) and in the present study was 0.77.

#### Mediating Variable

Social resources were measured using the six-item Lubben Social Network Scale (LSNS-6). The questions evaluate the frequency of contact and quality of kin and nonkin relationships. Score ranges from 0 (high isolation/few social resources) to 30 (low isolation/many social resources). The six-item scale has a reported alpha coefficient of 0.8 (Lubben et al., 2006) and in the present study was 0.74. For data visualization only, as determined in previous studies participants scoring less than 12 were categorized as socially isolated (Lubben et al., 2006).

#### Moderating Variable

Cognitive impairment was assessed by the Mini-Mental State Examination (MMSE). Items comprising the MMSE capture orientation, recall, attention, calculation, language, and visual construction (Folstein, Folstein, McHugh, & Fanjiang, 2000). MMSE scores range from 0 to 30 with higher scores representing greater cognitive capacity (lower impairment). The MMSE score is used in the statistical analysis, however, to represent severity of cognitive impairment graphically (Figure 4), we grouped scores according toSachdev and colleagues (2015): <23 (moderate to severe), 24–27 (mild), and 28–30 (intact or no significant impairment).

#### Covariates

Demographic covariates used in the analysis were gender (male/female), age (scale data), number of years of full time education, marital status (married versus not married), care setting (living in a care home or hospital versus not), and area deprivation (Townsend Index of Deprivation). The Townsend Index of Deprivation uses variables derived from the U.K. census on unemployment, overcrowded households; car/van ownership and home ownership. The score is calculated for Lower Super Output Areas (a geographical locale which contains on average 1,500 individuals, but varies depending upon population density). A greater score implies a greater degree of area deprivation (Townsend, Phillimore, & Beattie, 1988). Analysis used quintiles of the Townsend Index.

### Analytical Procedure

Descriptive statistics were produced for all variables (Table 1). Correlation analysis examined covariation between all variables in the model (Table 2). Using mediation, we tested whether social resources mediated the effects of disability on loneliness. Firstly, we determine the individual mediating effects of social resources after controlling for age, gender, education, marital status, care setting, and area deprivation. Secondly, we performed moderated-mediation analysis through construction and estimation of a conditional process model (Hayes, 2012a). Building on the mediation model, we test the moderating effects of cognitive impairment on the model’s “a” and “b” paths. In this moderated-mediation model we estimated the extent to which any indirect effect of disability on loneliness through the mediator “social resources” depends on the moderator “cognitive impairment.”

**Table 1.** Descriptive Statistics for Dependent, Independent, Mediating, and Moderating Variables

	*M*	*SD*	Range
Townsend disability score	2.4	1.38	0–4
Social resources (LSNS-6)	15.4	5.90	0–30
Cognitive impairment (MMSE)	26.9	3.02	8–30
Loneliness	1.0	1.18	0–6

**Table 2.** Correlation Analysis

		1	2	3	4	5	6	7	8	9	10
1	Disability		.38**	.18**	−.20**	−.19**	.14**	.11**	−.18**	−.34**	.14**
2	Age			.06**	−.14**	−.29**	.14**	.02	−.17**	−.34**	.06**
3	Gender				−.03	−.26**	.03	.04*	.03	−.08**	.04*
4	Education					.07**	−.04*	−.12**	.13**	.26**	−.00
5	Marital status						−.09**	−.12**	.09**	.17**	−.14**
6	Care setting							.03	−.07**	−.18**	.04*
7	Area deprivation								−.18**	−.34**	.14**
8	Social resources									.22**	−.34**
9	Cognitive impairment										−.11**
10	Loneliness										

*p < .05. **p < .01.

We used*PROCESS*, a computation procedure for SPSS developed byHayes (2012a) to implement mediation and moderated-mediation analysis to test the analytical models (Models 4 and 58:Hayes, 2012b). The bootstrap estimates were based on 5,000 random resamples of the data. We used 95% bias corrected and accelerated (BCa) confidence intervals (CI) to determine significant mediation effects. A variance inflation factor (VIF) was calculated for each predictor in the models, with values greater than 10 indicating high levels of multicollinearity (Hair, Anderson, Tatham, & Black, 1995).

## Results

### Descriptive Statistics

Around one quarter of the sample (*n* = 839, 25.3%) scored in the range of 2–6 on the loneliness scale, identifying this proportion of the sample as lonely (De Jong Gierveld & Van Tilburg, 2006). A similar proportion of the sample (*n* = 890, 26.9%) were identified as isolated, scoring below 12 on the Lubben Social Network Scale.

Bivariate correlation showed that the disability was significantly associated with all covariates, the proposed mediator (social resources), moderator (cognitive impairment), and loneliness. With regard to the covariates, greater levels of disability were associated with greater age, women, lower education, not currently married, and living in a care setting or a deprived area (Table 2). Overall, greater levels of disability were associated with poorer outcomes. The analysis supports Hypothesis 1 (that greater disability will be associated with greater levels of loneliness) and Hypothesis 2 (disability will have a negative influence on social resources). The VIF value for all predictors was <2, indicating that there was not a high degree of multicollinearity (Hair et al., 1995).

**Figure 2.**
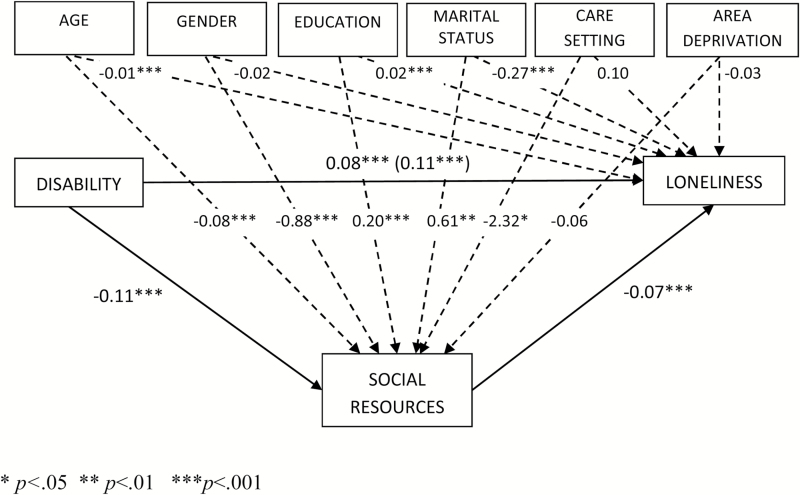
Statistical mediation model indicating the beta coefficients for disability, social resources (mediator), controls, and loneliness for Hypothesis 3.

Some control variables had a significant effect on social resources and loneliness (Figures 2 and3). Greater age was associated with fewer social resources and greater loneliness, while conversely, those that were married had greater social resources and lower levels of loneliness. A greater number of years in full time education was associated with greater social resources, but also with higher levels of loneliness. In the mediation model, men had fewer social resources and lower levels of loneliness than women. However, once cognitive impairment was included in the moderated-mediation model, men had significantly more social resources than women and there were no significant differences between men and women in levels of loneliness. Care setting was only significant in the mediation model demonstrating that older people living in a supported environment had lower levels of social resources than those living elsewhere. However, this association was accounted for by cognitive impairment, as in the moderated mediation model there was no significant association between the care setting and either social resources or loneliness.

**Figure 3.**
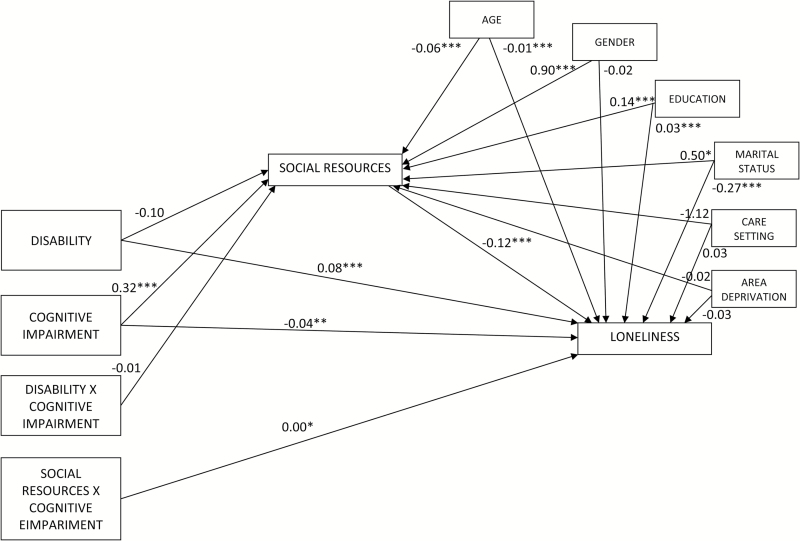
Statistical moderated-mediation model indicating the beta coefficients for disability, cognitive impairment (moderator), social resources (mediator), controls, and loneliness for Hypotheses 4 and 5.

### Mediation Analysis

Figure 2 shows the effect of disability on the mediator (“a” path) and the mediator’s effect on loneliness (“b” path) partialing out the effect of disability (and correcting for control variables). The total effect of disability on loneliness is significant (*c* = 0.11,*p* < .001), as is the direct effect (*c* ^1^ = 0.08,*p* < .001) although the strength of the association is weaker. By examining the 95% BCa CIs, we demonstrate that Hypothesis 3 is supported: disability has a significant indirect effect on loneliness through the mediating variable social resources $(\hat{a}\hat{b}$ = 0.34 [95% CI .023, .045]). Although disability has a negative effect on social resources, greater levels of social resources decrease loneliness. Thus, the mediating variables decreases the effect of disability on loneliness, weakening (rather than attenuating) the direct relationship between disability and loneliness.

### Moderated-Mediation Analysis

Turning to the moderators’ impact on the mediation model, cognitive function had a significant relationship with the mediator: greater cognitive impairment was associated with fewer social resources. Analysis of variance demonstrated significant difference in mean scores,*F*(3311, 2) = 63.28,*p* < .001 between those with moderate to severe cognitive impairment (*M* = 12.04,*SD* = 6.33) mild to moderate impairment (*M* = 13.85,*SD* = 5.67) and no significant impairment (*M* = 16.01,*SD* = 5.79). Cognitive impairment had greater effect on social resources than disability, consequently the path between disability and social resources is no longer significant (Figure 3). Although cognitive function is significantly associated with social resources, the interaction between disability and cognitive function is not, suggesting that Hypothesis 4 is not supported. As cognitive impairment does not exacerbate the link between disability and social resources we cannot reject the null hypothesis.

Considering the model’s “b” path, social resources have a significant impact on loneliness with greater levels of social resources decreasing loneliness. Cognitive function also has a significant impact on loneliness, whereby greater levels of cognitive impairment increase loneliness. Cognitive function also moderates the effect of social resources on loneliness. In this respect, greater cognitive impairment weakens the association between social resources and loneliness. Operationalizing cognitive impairment as “high” (1 SD below sample mean) and “low” (1 SD above sample mean) shows that for MMSE the conditional indirect effects on loneliness was −.072,*SE* .005 in the “high” MMSE condition and was statistically significant*t*(3314)= −15.803,*p* < .001, 95% CI (−.08, −.06). In the “low” condition the effect was weaker −.059,*SE .*005, but still statistically significant*t*(3314) = −12.755,*p* < .001, 95% CI (−.07, −.05). This shows that the moderating effects of cognitive impairment decreased at greater levels of functioning.

**Figure 4.**
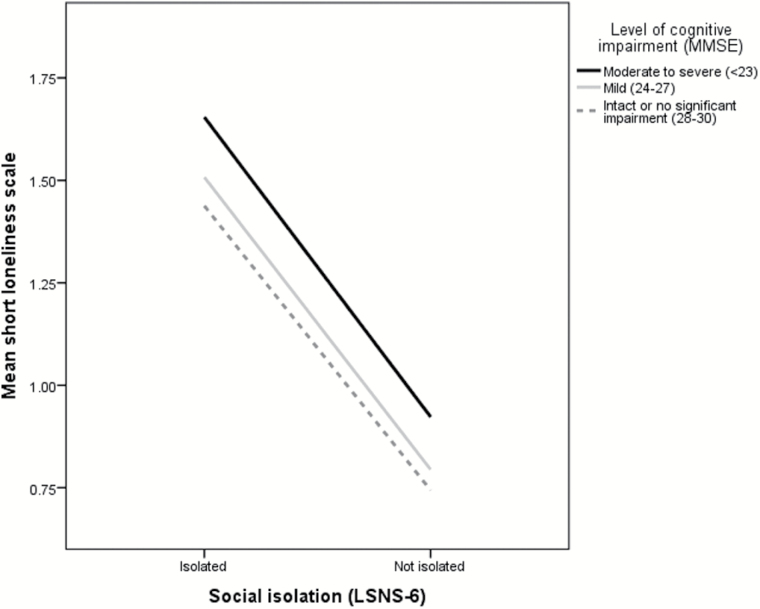
Loneliness and isolation at different levels of cognitive impairment.

For data visualization purposes,Figure 4 demonstrates the interaction effect by examining mean loneliness scores by three levels of cognitive impairment, and for those that were isolated versus those that were not. The data indicate that participants with moderate to severe cognitive impairment experienced more loneliness than participants with better cognitive function, and those with mild to moderate cognitive impairment felt lonelier than those with no significant impairment. Across all three groups, loneliness is greater for those that are isolated compared to those that are not, and after taking into account levels of social resources, for those with greater levels of cognitive impairment (i.e., regardless of whether they were isolated or not). Hypothesis 5 is supported as cognitive function had a moderating influence on the model’s “b” path: cognitive impairment amplified the experience of loneliness through the mediating variable social resources.

## Discussion

Loneliness is often over-estimated in the older population and perceived to be a greater problem than it is (Dykstra, 2009). Loneliness was experienced by one quarter of older people in the study which is similar to the proportions observed elsewhere, ranging between 20% and 30% in Europe (Dykstra, 2009), 19% in the United States (Theeke, 2009), and 31% in Australia (Steed, Boldy, Grenade, & Iredell, 2007). Often older people consider a good social life to be the most important aspect of subjective well-being (Douma, Steverink, Hutter, & Meijering, 2015). Consequently, improving social engagement and decreasing loneliness has become a common driver of health and social policies.

The ICF (WHO, 2001) model of disability acknowledges that social-structural and psychosocial factors such as social support (Verbrugge & Jette, 1994), psychological resources (Femia, Zarit, & Johansson 2001), and discrimination (Owens, 2015), influence outcomes. The ICF model has similarities to the cognitive discrepancy model, especially with the addition of the “social-cultural and social structural context” as proposed by the authors. While some research has looked at the extent to which the social environment impacts on people with dementia in care settings (e.g., the work on person-centered care;Kitwood, 1988) there is little evidence about its impact on people with less severe cognitive impairment living in the community.

The hypothesis that cognitive impairment would amplify difficulties associated with disability was not supported. However, we demonstrated that cognitive impairment had a greater effect on social resources than disability. Elsewhere, experts have suggested that measures of instrumental activities (similar to the modified Townsend Index of disability used in this study) do not capture changes in behaviors observed, for example, in people with mild cognitive impairment (Burton, Strauss, Bunce, Hunter, & Hultsch, 2009). In our study, the average level of social resources for participants experiencing moderate to severe cognitive impairment was greater than the clinical cut-point for social isolation (Lubben et al., 2006). Our analysis cannot determine whether biological manifestations of cognitive impairment or social-structural factors impact on social resources. However, our analysis suggests that the association between cognitive impairment and social resources is incremental and has an influence at both low and appreciable levels.

Response to and awareness of cognitive impairment and the resulting maintenance or adaption of self-identity varies from person to person. Similarly we proposed that the moderating influence of cognitive impairment on the pathway between social resource and loneliness may take on a variety of forms. Our analyses were based on the premise that self-identity and social comparison are attributions that will be influenced by cognitive processes (Figure 1). Research in Ireland found that depressive symptoms interfere with judgments about the adequacy of social interaction and have an indirect moderating effect on loneliness (Burholt & Scharf 2014). WhileBurholt and Scharf (2014) suggested that adjusting one’s expectations regarding*desired* social relations is more difficult to achieve for those with depression, we propose that there are slightly different mechanisms at work for older people with cognitive impairment. In this respect cognitive impairment has a moderating influence on the judgment that a person makes concerning the adequacy of the quality and quantity of social relations, but there are individual differences in moderating responses (Buunk & Mussweiler, 2001). More specifically, we suggest that (a) the social context influences social comparison and (b) there are biological and neurological influences that limit self-knowledge and social comparison.

In describing the cognitive discrepancy theoryPerlman and Peplau (1981) pointed to the potential for cognitive processes to modulate the loneliness experience. They suggested that labelling, causal attributions, social comparison, and perceived control could influence loneliness. Our results could be interpreted as incorporating elements of each of these. For example, internalization or identification with negative images of cognitive decline and social withdrawal could contribute to self-perceptions of loneliness. Elsewhere research has found minority groups are at risk of accepting and internalizing negative societal values which influences social engagement and impacts on loneliness (Kim & Fredriksen-Goldsen, 2014). Furthermore, attributing loneliness to cognitive impairment may situate this beyond the individual’s control resulting in anticipation of prolonged loneliness (Wangler, 2014). According to the original social comparison theory (Festinger, 1954) “choice” plays a major role in how people contrast their situation to others. When faced with a threat such as ill health,Wills (1981) suggested that downward comparison (comparing one’s situation to others in a “worse” situation) could lead to improved self-esteem and wellbeing. Indeed, research has demonstrated this effect for older people with chronic disease (Arigo, Suls, & Smyth, 2014). However, our results suggest that downward social comparison is not a significant modulator of loneliness for those with cognitive impairment. Alternatively, a social identity that is predominantly constructed through negative attitudes, norms and collective discourses may result in an upward comparison (comparing one’s social situation to those “better off”) and affects the experience of loneliness.

Making a judgment about the adequacy of social resources assumes that a person has self-knowledge to evaluate their situation against others (Buunk & Mussweiler, 2001). In the presence of anosognosia a person may rely upon a self-identity that is based on past behavior before the onset of cognitive impairment (Ruby et al., 2007). Consequently, the desire for a certain level of social resources may be unrealistically optimistic and outstrip the objective assessment of social interactions. In this respect, cognitive dissonance between actual and desired social relation may be the result of an unwitting temporal social comparison with an earlier self-identity.

### Limitations and Future Directions

The research outlined in this article was conducted in Wales, UK, and the models should be tested with data from other countries to ascertain the applicability in other cultural contexts. The mediation and moderated-mediation models provide only two examples of pathways to loneliness, and other models may fit the data better. For example, the models might be improved by including personality traits, sense of control or resilience that may predispose people to one form of social comparisons over another (Buunk & Mussweiler, 2001). The (currently) cross-sectional nature of CFAS Wales data means that we cannot be sure of the direction of causality, and as noted in the introduction other studies have found bidirectional relationships. However, CFAS Wales is a longitudinal study, and future waves of data will provide an opportunity to test these causal pathways with more conviction.

We have suggested that structural barriers are partially responsible for the negative impact of cognitive impairment on social resources. However, we acknowledge that further research is required to establish the extent to which noncognitive symptoms of cognitive impairment (such as apathy, fear, anger;Kasper, Oswald, Wahl, Voss, & Wettstein, 2012) are related to social structural barriers within society that impact on social interaction. Very little research has paid attention to the day-to-day consequences of cognitive impairment, environmental incongruence, and resulting behaviors.

Finally, our model is only a partial representation of cognitive discrepancy theory as it does not include a measure of expectations for social interaction. Our model suggests that cognitive impairment moderates the relationships between social resources and loneliness and we have speculated that there may be distinct ways in which this operates according to cognitive resource (e.g., through internalization of negative beliefs or anosognosia). Consequently, qualitative research is warranted to explore the relationship between social comparison and loneliness at different levels of cognitive impairment.

### Implications

Recent critiques of the social model of disability suggest that the role of biology is often downplayed in terms of its impact on the lives of people experiencing disability (Anastasiou & Kauffman, 2013). While our focus has not been on health outcomes, we acknowledge that intervening to decrease loneliness may have a wider public health benefit. Research has demonstrated a relationship between loneliness and poor health outcomes such as an increased risk of mortality (Holt-Lunstad, Smith, Baker, Harris, & Stephens, 2015) or fatal and non-fatal suicide behaviors (Fässberg et al., 2012). Moreover, in highlighting social-structural and psychosocial factors associated with cognitive impairment that may influence social resources and loneliness, we are not implying that the models or theories that demonstrate social interaction or loneliness influence cognitive function are wrong: we are not disputing neurological and biological explanations for deterioration in social skills (Holwerda et al., 2012). Instead, we have raised hypotheses about alternative (additional) mechanisms whereby cognitive functioning influences wellbeing outcomes (loneliness) for older people. As an alternative, we have considered the ways in which the social environment impacts on people lives. Consequently, the implications for our research are based on the potential for sociocultural, social structural and psychosocial interventions.

There are two main messages that arise from our research: (a) loneliness may be influenced by the social environment (e.g., internalization of negative images of cognitive impairment, or discrimination) and interventions focused on these elements may have a positive impact and (b) tackling loneliness requires joined up theorising and integrated research from biologists, neurologists, psychologists, environmental geographers, through to social policy analysts. A recent systematic review of interventions to alleviate loneliness identified four primary strategies that are generally adopted: (a) improving social skills, (b) enhancing social support, (c) increasing opportunities for social interaction, and (d) addressing maladaptive social cognition (Masi, Chen, Hawkley, & Cacioppo, 2011). The authors of the review concluded that correcting maladaptive social cognition (e.g., through cognitive behavior therapy) offers the best chance for reducing loneliness. However, there are no robust evaluations of the effects of community-based policy interventions that aim to improve the social environment and indirectly impact on loneliness. Arguably any recommendations arising from meta-analyses will be skewed because of the domination of the medical model of loneliness in the published literature, and the selection of studies focusing specifically on decreasing loneliness as a primary outcome.

Globally, the notion of dementia supportive communities has gained momentum.*Dementia positive* policies advocate for positivity toward people with cognitive impairment, manifest in attitudes, beliefs, communication, and behaviors (Lin & Lewis, 2015). The attitudes of staff and public in facilities that are considered part of the normal routine of daily living (such as shops, galleries, and sport centers) can influence whether or not a person continues to engage in social activities (Woods, 2012). Attitudes are amenable to change and the media has a large role to play in the representation of aging and health (Wangler, 2014). Ultimately, changing cultural attitudes has the potential to impact on self-identity and the perception of social futures for those with cognitive impairment. However, further research is required to assess the impact of dementia positive policies (Lin & Lewis, 2015). The lack of agreement concerning definitions of the concepts of dementia-friendly, dementia-capable, or dementia-positive communities and the range of plans, policies, strategies, or frameworks that may comprise interventions to promote social inclusion poses inherent difficulties for systematic reviewers. As an alternative, realist synthesis, which attempts to provide an explanatory analysis of how and why complex social interventions work (or not) in particular contexts could contribute to robust evaluation (Wong, Greenhalgh, Westhorp, Buckingham, & Pawson, 2013).

Despite the potential for cultural change to impact on the prevalence of loneliness, developing dementia supportive communities are not likely to impact on temporal comparison and the experience of loneliness due to anosognosia. An integration of the different approaches to loneliness is required in order to stimulate critical reflection on the implications and limitations of our knowledge. Biological or neurological perspectives alert us to the potential for further decline in cognitive function based on stress, education, or lack of intellectual social engagement. A critical gerontological perspective highlights the ways in which wider social structures (dis)empower people with cognitive impairment and lead to exclusion from social resources or impacts on the social construction of aging, cognitive impairment, and dementia. Psychosocial perspectives offer insight into the way in which the individual with cognitive impairment may view themselves (the sense of self) and may be influenced by social norms and stereotypes, or through a temporal social comparison with an “earlier” sense of self. Ultimately, we propose that these approaches are inter-related and in order to develop effective interventions the theorizing behind the relationships between disability, social resources, loneliness, and cognitive impairment needs to be explicit. Understanding the link between the individual, the social-cultural and social structural environment, social interaction, cognitive processes (such as social comparison), and loneliness can provide a generalizable framework to inform the delivery and development of multimodal intervention strategies. In the long run, new research integrating theoretical approaches could inform interventions tailored to meet the multifarious causes of loneliness.

## Funding

This work (CFAS Wales study) was supported by the Economic and Social Research Council (RES-060-25-0060) and Higher Education Funding Council Wales as “Maintaining function and well-being in later life: A longitudinal cohort study”.
